# Multicomponent Exercise Improves Hemodynamic Parameters and Mobility, but Not Maximal Walking Speed, Transfer Capacity, and Executive Function of Older Type II Diabetic Patients

**DOI:** 10.1155/2018/4832851

**Published:** 2018-02-14

**Authors:** Hélio José Coelho Junior, Iris Callado Sanches, Marcio Doro, Ricardo Yukio Asano, Daniele Jardim Feriani, Cayque Brietzke, Ivan de Oliveira Gonçalves, Marco Carlos Uchida, Erico Chagas Capeturo, Bruno Rodrigues

**Affiliations:** ^1^School of Physical Education, University of Campinas, Campinas, SP, Brazil; ^2^Human Movement Laboratory, São Judas Tadeu University (USJT), São Paulo, SP, Brazil; ^3^School of Arts, Sciences and Humanities, University of São Paulo, São Paulo, SP, Brazil; ^4^Center of Health Sciences, University of Mogi das Cruzes, Mogi das Cruzes, SP, Brazil

## Abstract

The present study aimed to investigate the effects of a 6-month multicomponent exercise program (MCEP) on functional, cognitive, and hemodynamic parameters of older Type 2 diabetes mellitus (T2DM) patients. Moreover, additional analyses were performed to evaluate if T2DM patients present impaired adaptability in response to physical exercise when compared to nondiabetic volunteers. A total of 72 T2DM patients and 72 age-matched healthy volunteers (CG) were recruited and submitted to functional, cognitive, and hemodynamic evaluations before and after six months of a MCEP. The program of exercise was performed twice a week at moderate intensity. Results indicate T2DM and nondiabetic patients present an increase in mobility (i.e., usual walking speed) after the MCEP. However, improvements in maximal walking speed, transfer capacity, and executive function were only observed in the CG. On the other hand, only T2DM group reveals a marked decline in blood pressure. In conclusion, data of the current study indicate that a 6-month MCEP improves mobility and reduce blood pressure in T2DM patients. However, maximal walking speed, transfer capacity, and executive function were only improved in CG, indicating that T2DM may present impaired adaptability in response to physical stimulus.

## 1. Introduction

The aging process leads to several alterations in the functioning of some, if not all, physiological systems, collaborating with the development of chronic diseases, such as Type 2 diabetes mellitus (T2DM). Indeed, over 11 million older people presented a clinical diagnosis of T2DM in the United States of America in 2014, representing around 25% of this population [1].

The progression of T2DM leads to the development of poor outcomes, such as diabetic peripheral neuropathy, which is known by its deleterious effects on muscle architecture and functioning, reducing muscular functionality (e.g., mobility, transfer capacity) [2–4].

Several studies have indicated that T2DM patients have a poor cognitive status when compared to age-matched healthy volunteers [5–9]. Although cognition is formed by several capacities (e.g., memory, attention, and inhibition), most authors have highlighted the key role of executive function in the management of T2DM since T2DM patients with executive dysfunction present a high risk to medical assistance [8]. These data deserve concern, mainly in older adults, because the Pan American Health Organization (PAHO) stated independence (i.e., physical function) and autonomy (i.e., cognitive capacity) as two factors corresponding to the concept of health in this population [10].

In addition, the physiopathology of T2DM is strongly associated with a number of alterations in the mechanisms of blood pressure control (e.g., elevated activity of the renin-angiotensin system, autonomic dysfunction), which may trigger the development of high blood pressure in these patients [11, 12]. Indeed, it is worth mentioning that populational data have suggested that more than 50% of the T2DM patients present a clinical diagnosis of hypertension, consequently increasing the poor prognosis in this population [13].

Lastly, T2DM is accompanied by a number of other alterations in the cerebral (i.e., low cerebral blood flow) and muscular (i.e., altered muscular protein metabolism, increased oxidative stress) apparatus [14–19]. In addition to impairing the homeostasis of the organic system, these aforementioned features may reduce the adaptability of T2DM patients in response to physical stimulus (e.g., exercise training [ET]).

Interestingly, an inability to adapt in response to physical exercise may be one more negative factor in the context of T2DM since ET has been shown to improve morphofunctional, cognitive, and hemodynamic parameters of patients with different conditions and could be used to reverse or even stabilize the clinical symptoms of T2DM patients.

In fact, aerobic exercise, for example, is widely known for its key role in the control of blood pressure of healthy and hypertensive patients [20]. Resistance training is suggested as an excellent approach to cause muscular hypertrophy and improve neuromuscular parameters, such as muscle strength and power [21–24]. Moreover, a systematic review of randomized controlled trials indicated that improvements in balance—which is essential for the performance of functional tests, such as* Timed “Up and Go” [TUG]—*are dependent on postural instability, suggesting that balance training should be added in programs aimed at improving this physical capability [25].

Despite the beneficial effects of these interventions in the various above-mentioned domains, some organizational and operational difficulties appear when a long-term ET program is supposed to adequately provide these regimes of physical exercise alone within a periodization period for older T2DM patients. In this sense, multicomponent exercise program (MCEP) emerges as an alternative kind of ET able to combine different exercise regimes in the same exercise routine, thereby not requiring sessions of physical exercise with long duration while developing several physical capacities and skills [26].

Indeed, recently, we demonstrated that a 6-month MCEP was able to increase balance and mobility in normotensive and hypertensive patients [27]. However, the hemodynamic changes in response to the exercise program were not investigated, and our sample was composed of hypertensive and normotensive patients without T2DM. Therefore, the effects of MCEPs on T2DM patients still remain to be elucidated. To the best of our knowledge, just one investigation studied the effects of a MCEP on T2DM patients [28], but the findings were based on subjective methods (i.e., questionnaires).

Therefore, the present study aimed to investigate the effects of a 6-month MCEP on functional, cognitive, and hemodynamic parameters of older T2DM patients. In addition, we investigated if T2DM patients presented impaired adaptability in response to physical exercise when compared to age-matched healthy volunteers.

## 2. Materials and Methods

**Figure 1:**
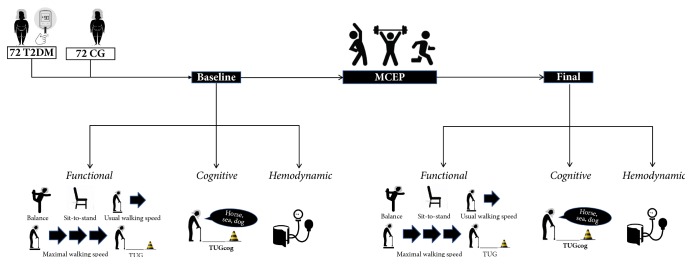
Experimental design of the current study.

The findings present in the current study are part of a larger study which aimed to investigate the effects of MCEPs on functional, cognitive, and hemodynamic parameters of community-dwelling older adults with different chronic conditions (e.g., hypertension). The findings regarding hypertensive patients have been previously published by our group [27]. In the present trial, we used a single-group quasi-experimental design to determine the effects of a 6-month MCEP in the functional (i.e., mobility, maximal walking speed, lower limb muscle strength, balance, and transfer capacity), cognitive (i.e., executive function), and hemodynamic parameters of older Type 2 diabetes mellitus (T2DM) patients. Secondarily, to investigate if T2DM patients present impaired adaptability to ET, results were compared with a control group (CG) composed of age-matched healthy volunteers, who were submitted to the same protocol of MCEP. Therefore, volunteers were submitted to functional, cognitive and hemodynamic evaluations before and after a 6-month MCEP (Figure 1). We followed the methods of Coelho Junior et al. (2017) [29].

All volunteers signed the informed consent form and completed all measurements. This study was approved by the Research Ethics Committee of the University of Campinas (UNICAMP). This study was developed in accordance with the Declaration of Helsinki and according to Resolution 196/96 of the National Health Council.

## 3. Subjects

The sample consisted of community-dwelling older untrained volunteers from Poá, São Paulo, Brazil. Participants were recruited by convenience from a specialized healthcare center for older adults. Volunteers were asked verbally by the medical team and researchers about their participation in the study. Eligibility criteria for this study were based on the presence of a clinical diagnosis of T2DM and being aged ≥ 60 years. Subjects in the control group (CG) did not present a clinical diagnosis of T2DM. Patients of both sexes were accepted in the study. Patients who were absent in ≥3 subsequent sessions of exercise and unable to perform the TUG cognitive test [30] and presented a clinical diagnosis of hypertension, cardiovascular disease, cerebrovascular disease, neurological or psychiatric disease, pulmonary disease, musculoskeletal disorders, comorbidities associated with greater risk of falls, and any sign of orthostatic hypertension and/or labyrinthitis were excluded. One hundred forty-four participants (72 T2DM and 72 age-matched healthy [CG]) completed all evaluations and were eligible to participate in the present study.

Subsequently, CG and T2DM groups were created according to the previous clinical diagnosis of T2DM. It should be stressed that the diagnosis of T2DM was made according to the guidelines of the* Brazilian Society of Diabetes*.

## 4. Evaluations

The participants were instructed to refrain from any exhausting activity for a period of 96-h before testing and drinking alcoholic and caffeinated beverages 24 h before testing. Subjects were requested to maintain their food intake during the entire protocol (i.e., ~6 months). Baseline evaluations (i.e., before) were performed 5 days (i.e., 120 hours) before the beginning of the MCEP. Likewise, the final evaluations were performed on the fifth day after the last exercise session.

### 4.1. Functional Parameters

Two experienced researchers applied each test. While one was responsible for detailing the operational procedures, demonstrating the test before the evaluation, quantifying the evaluation time, and evaluating the motor gesture, the other ensured the safety of the participant. After the end of the explanation and before the start of the tests, volunteers performed a familiarization trial to ensure the understanding of the test. The tests were performed twice, and the best result obtained was used in the final analysis. The tests were distributed in a room as stations and were performed in a circuited fashion one after the other. A one-minute interval between trials was provided. Verbal encouragement was provided throughout the tests to ensure that volunteers achieved the best possible performance without compromising safety. During TUG, walking speed test at maximal pace, and sit-to-stand tests researchers provided stimulus such as the following: “*Come on, faster!”*;* “A little more!”*; and;* “Let's go!” *During one-leg stand, verbal encouragement was provided to keep the participant focused on the test. Therefore, the volunteers were stimulated with the sentences: “*Focus! Keep your posture!”* and “*Very good!”* The present protocol has been published by our group elsewhere [29].

#### 4.1.1. Sit-to-Stand Test

Volunteers were requested to rise from a chair five times as quick as possible with arms folded across the chest. The stopwatch was activated when the volunteer raised their buttocks off the chair and was stopped when the volunteer was seated back at the end of the fifth stand.

#### 4.1.2. One-Leg Stand Test

The one-leg stand test was performed with the volunteers standing in a unipodal stance with the dominant lower limb, the contralateral knee remaining flexed at 90°, the arms folded across the chest, and the head straight. A stopwatch was activated when the volunteer raised their foot off the floor and was stopped when the foot touched the floor again. The maximum performance time was up to 30 seconds, considered the best test result.

#### 4.1.3. Three-Meter Usual and Maximal Walking Speed Test

To measure 3-meter walking speed, a 3-meter walking speed test was performed. Volunteers were required to walk a distance of five meters at their usual and fastest possible cadences (without running). Before the evaluation, both feet of each volunteer were to remain on the starting line. The measurement was initiated when a foot reached the one-meter line and was stopped when a foot reached the four-meter line. The one-meter intervals at the beginning and end were used to avoid early acceleration and/or deceleration.

#### 4.1.4. Timed “Up and Go” (TUG) Test

Transfer capacity was evaluated by the Timed “Up and Go” (TUG) test. The TUG test involves getting up from a chair (total height: 87 cm; seat height: 45 cm; width: 33 cm), walking three meters around a marker placed on the floor, coming back to the same position, and sitting back on the chair. The subjects started the test and wore their regular footwear, with their back against the chair, with arms resting on the chair's arms, and with the feet in contact with the ground. A researcher instructed the volunteers to, on the word “go,” get up and walk as fast as possible without compromising safety in the demarcation of three meters on the ground, turn, return to the chair, and sit down again. Timing was started when the volunteer got up from the chair and was stopped when the participant's back touches the backrest of the chair. A stopwatch (1/100 second accuracy) was used for time evaluation, and a longer time taken to perform the test indicates a lower performance.

### 4.2. Executive Function (EF)

#### 4.2.1. TUG Cognitive Test

TUG cognitive test was accomplished to evaluate EF. The motor task of this test is similar to the conventional TUG. However, a cognitive task (verbal fluency, animal category) must be accomplished during the motor task. Therefore, after the signal of the evaluator, the volunteer performed the route—stand up from the chair, walk three meters, turn around, walk three meters back, and sit down again—naming as many animals as he/she could remember. This task was performed aloud, allowing the evaluators to confirm if the volunteers were, in fact, accomplishing the task. The time expansion to perform the task was recorded to evaluation [30]. Briefly, the number of animals mentioned during the test was not recorded. However, one researcher was responsible for ensuring that the volunteers kept naming the animals throughout the whole test.

### 4.3. Hemodynamic Measurements

The procedures for measurement of blood pressure were adapted from the VII Joint National Committee on Prevention, Detection, Evaluation, and Treatment of High Blood Pressure (JNC7) [31]. In summary, volunteers remained in a sitting position on a comfortable chair for 15 minutes in a quiet room. After this period, an appropriate cuff was placed at approximately the midpoint of the upper left arm (heart level). An automatic, noninvasive, and validated [32] arterial blood pressure monitor (Microlife-BP 3BT0A, Microlife, Widnau, Switzerland) was used to measure systolic blood pressure (SBP), diastolic blood pressure (DBP), and heart rate (HR). During blood pressure recording, volunteers remained relaxed in the sitting position, with parallel feet at one shoulder width, both forearm and hands on the table, supinated hands, and back against the chair, without move or talk. The volunteer did not have access to blood pressure values during measurement. The evaluation lasted approximately 80 seconds and was performed three times with one minute of rest among the measurements. The mean of measurements of each volunteer was used in the final analysis. Mean arterial pressure (MAP), double product (DP), and pulse pressure (PP) were evaluated according to the following equations: MAP = [SBP + (2 *∗* DBP)]/3, DP = SBP *∗* HR, and PP = SBP – DBP.  The size of the arm cuff was selected after measuring the arm circumference of each participant (Sanny, São Paulo, Brazil). All volunteers were evaluated within the first month after the update of the medical records.

## 5. Multicomponent Exercise Program (MCEP)

The MCEP was performed twice a week, on nonconsecutive days, during 26 weeks at the fitness center of an institutional center for elderly care and living (Centro de Convivência do Idoso [CCI]), Poá, Brazil [33]. The program was designed to offer exercises that would mimic activities of daily live (ADL) gestures, thereby inducing neuromuscular adaptations to maintain the subjects capable of performing the ADL. Twelve different exercises stations composed each exercise session. The structure of each exercise session was defined by the sequence of one functional exercise followed, immediately, by a brief walk. Therefore, exercise session was composed of approximately 12 minutes of functional exercises, 24 minutes of walk, and 12 minutes of rest. Approximately 50 patients composed each session of exercise. A physical trainer professional with larger expertise on exercise training to older people supervised all sessions (Gonçalvez IO). Volunteers were instructed to avoid the Valsalva maneuver during the performance of exercises.

The functional exercises were changed during the whole program. However, they always represented ADL with a large necessity of the activity of the lower limbs, for example, stand and seat from the chair, pick up a weight off the floor and put on top of a structure, and transfer a weight from one place to another. Balance and proprioception exercises also composed functional exercises, as one-leg stand. At most three balance and/or proprioception exercises were used in each session. To complete the list of physical exercises, upper limbs resistance exercises were added.

All functional exercises were performed for one minute. The brief walk was performed for two minutes. Thus, after the end of each functional exercise, volunteers must walk from one point to another (30 m), around a cone, come back to the initial line (30 m), and start the path again until completing the two minutes. A rest interval of 60 seconds was adopted between the stations.

## 6. Exercise Intensity Control

The control of exercise intensity was accomplished by an 11-point scale (CR-10) to measure the perceived exertion (RPE) [34], which was used to ensure that volunteers performed the exercises in the aimed intensity. This scale is composed of eleven numbers (i.e., 0, 1, 2, 3, 4, etc.) and eight descriptors (i.e., rest; very, very easy; easy; moderate; somewhat hard; hard; very hard; maximal), which represents the perception of effort of the subject in front of an exercise load. The higher the reported number, the greater the sensation of effort [34]. During the performance of functional—except for balance exercises—and resistance exercises, volunteers were instructed to maintain the physical activity intensity in 3–5—which represents moderate (i.e., 3), somewhat hard (i.e., 4), and hard (i.e., 5) descriptors. To that, a large picture of RPE scale (i.e., 4 meters high and 1.30 meters wide) was positioned on the wall in the gym's room. The increase in the exercise intensity was based on alterations in the cadence of the performance (i.e., faster), for functional exercises and walk. Moreover, for resistance exercises, volunteers could use elastic bands (EXTEX Sports, São Paulo, Brazil) and dumbbells to reach the intensity prescribed.

## 7. Statistical Analyses

Normality of data was tested using the* Kolmogorov-Smirnov* test. Baseline comparisons between the groups for age, morphology, and circumferences were performed using one-way analysis of variance (ANOVA) followed by Tukey's post hoc test. A two-way ANOVA followed by a Dunnett post hoc test was performed to identify differences among the different times of evaluations and treatments. Cohen's effect size *d* was calculated to assess the magnitude of the results. The effect size was classified according to Rhea, 2004 [35], for untrained volunteers. The level of significance was 5%  (*P* < 0.05) and all procedures were performed using the GraphPad Prism 6.0. (San Diego, California, USA).

## 8. Results

No adverse events occurred during the sessions of exercise or during any of the evaluations. The subjects were not absent for more than three sessions of physical exercise. Adherence to the physical exercise program was 100% (0 dropouts). This interesting result probably occurred because the present study was offered for older adults in need of public attention and the volunteers wait a long time to participate, so that we believe that most of them understood the importance of the exercise program in their lives. Indeed, before the beginning of the MCEP, some speeches were made in an attempt to make them understand the importance of the project. Moreover, it is possible to infer that the current MCEP was attractive to them, since it was composed of a mix of different exercise protocols, avoiding monotony. Other possible explanations for this rate of dropout are the kind of measurements that were performed—because we did not perform several evaluations or discouraging assessments (i.e., biopsy)—and the design of the program—because volunteers did not have to remain in a rigorously controlled state—and the exclusion criteria that were adopted since subjects were just excluded if they were absent from ≥3 subsequent sessions of exercise. Volunteers did not report any changes in food intake and or in the number/class of medications during the study.

Overall characteristics are shown in Table 1. BMI evaluation indicated that T2DM and CG presented an overweight and obese phenotype, respectively. However, statistical significant differences were not observed between the groups. Circumference values (i.e., WC, HC, WC/HC, and NC) are in line with BMI evaluation, since CG and T2DM presented a high cardiovascular risk. The hypothesis test indicated that T2DM presented a higher WC, HC, WC/HC, and NC when compared to CG.

**Figure 2:**
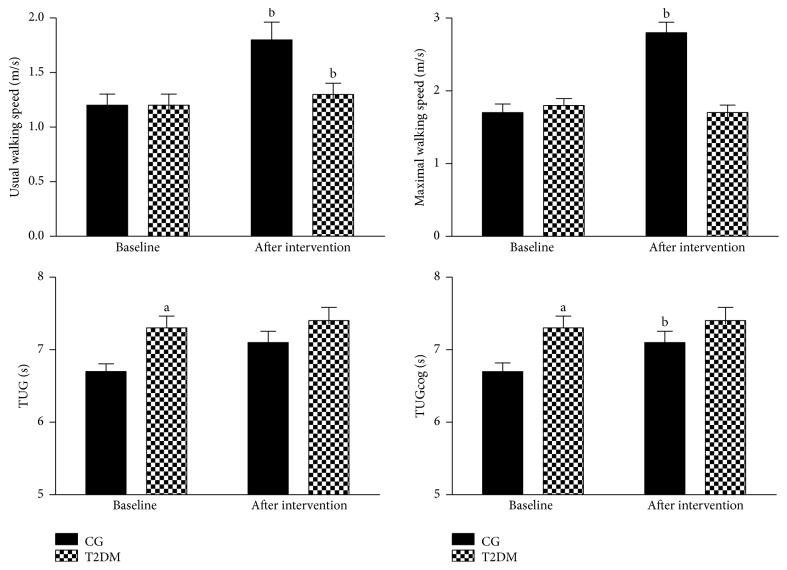
Functional and cognitive parameters. CG = control group; T2DM = Type II diabetes mellitus; TUG = Timed “Up and Go”; ES = effect size; ^a^*P* < 0.05 versus CG; ^b^*P* < 0.05 versus baseline.

Functional and cognitive parameters are shown in Table 2 and Figure 2. At baseline, T2DM presented higher TUG and TUGcog values when compared to CG, indicating an impaired performance in both tests compared to non-T2DM volunteers. There were no significant differences regarding the other parameters. A significant increase on usual walking speed was observed after the 6-month MCEP, regardless of T2DM. However, just CG displayed an improved performance in maximal walking speed and TUGcog (Figure 2). ES evaluation corroborates with the hypothesis test, since ES classification was always higher in the CG (i.e.,* small *and* moderate*) when compared to T2DM (i.e.,* trivial* and* small*) group (Table 2). Nevertheless, intergroup comparisons (i.e., Post and delta [Δ]) did not demonstrate significant differences between the groups after the MCEP. Lastly, sit-to-stand, one-leg stand, and TUG tests were not significantly altered in any of the groups after the exercise program.

Hemodynamic parameters are shown in Table 3. At baseline, T2DM presented higher SBP values when compared to CG, while the other hemodynamic parameters were similar between the groups. T2DM presented a significant decrease in SBP, DBP, and MAP after the MCEP; whereas CG did not show significant alterations. ES evaluation are in line with the hypothesis test, since ES classification was always higher in the T2DM (i.e.,* trivial and small*) when compared to CG (i.e.,* trivial*). The intergroup comparisons (i.e., Post and delta [Δ]) after the MCEP did not demonstrate significant differences between the groups.

## 9. Discussion

The main findings of the present study indicate that T2DM patients show reduced executive function and transfer capacity when compared to nondiabetic volunteers (i.e., CG). On the other hand, T2DM presented higher blood pressure values than CG. Our data indicate that a 6-month MCEP is able to improve mobility in T2DM and nondiabetic patients, while a significant increase in maximal walking speed, transfer capacity, and executive function was only observed in CG. Lastly, T2DM patients presented a marked decline in the hemodynamic parameters, which was not observed in CG.

Our results are in line with prior cross-sectional and prospective populational studies that reported a decreased executive function in T2DM patients [5–9]. In the study of Qiao et al. (2006) [6], authors observed that T2DM patients presented a poor executive function (overall and in the subdomains [visuospatial, working memory]) and global function scores when compared to patients without T2DM. These data deserve concern, since sufficient cognitive skills, mainly preserved executive function, are required for adherence and effectiveness of diabetes treatment because an elevated risk for hypoglycemia requiring medical assistance is observed in T2DM with cognitive dysfunction [8].

In addition, executive function seems to be associated with mobility capacity because an ability to process, integrate, and respond to multiple stimuli received during ambulation is necessary for a successful locomotion [36]. Indeed, executive dysfunction is associated with impaired gait and balance, which plays a key role in the TUG performance of healthy and cognitively impaired older adults [37]. Therefore, the reduced executive function presented by our T2DM patients may be associated with their impaired transfer capacity.

However, executive dysfunction is probably not the only factor responsible for the reduced TUG scores observed in the current study since T2DM progression synergistically with prolonged hyperglycemia exposure is known to result in diabetic peripheral neuropathy, which is associated with several deficiencies in the morphology and functioning of the skeletal muscle—such as denervation-reinnervation process and loss of motor axons and nerve excitability and conduction (i.e., demyelination)—resulting in accelerated muscle atrophy and fatigue and loss of muscle strength and power (i.e., muscle weakness), consequently reducing motor function (e.g., walking, climbing stairs) [2–4].

It is noteworthy that the current 6-month MCEP was not able to improve transfer capacity and executive function in T2DM, while a significant increase in mobility (i.e., usual walking speed) was observed. On the other hand, CG presented significant improvements in maximal walking speed, transfer capacity, and executive function. These data suggest that T2DM present a limited adaptability in response to physical exercise—at least on executive function and neuromuscular capabilities—when compared to age-matched healthy controls.

Findings of the present study are in line with other experiments, which demonstrated an increased mobility in T2DM patients after ET [for review see Cadore and Izquierdo, 2015 [38]]. However, only a few studies have been designed to investigate the effects of a MCEP in these variables, limiting our discussion.

In the trial of Baptista et al. (2017) [28], T2DM patients were submitted to a 24-month MCEP performed 3 days per week and composed of 30 minutes of aerobic exercise, 20 minutes of resistance exercise, and 20 minutes of balance and flexibility exercises. The program of exercise elicited significant improvements in physical functioning, but not in mental health. Nevertheless, it is worth mentioning that the findings of Baptista et al. (2017) [28] were based on a self-reported questionnaire (i.e., SF36), because the main objective of their study was to evaluate health related quality of life, strongly limiting further comparisons between our studies.

Therefore, in addition to the findings of Baptista et al. (2017) [28], our findings suggest that a MCEP composed of resistance, aerobic, and balance exercises performed at moderate intensity may improve mobility and the self-perception of mobility in T2DM patients.

Regarding the lack of significant alterations in the executive function, maximal walking speed, and transfer capacity observed in T2DM after the 6-month MCEP, some investigations support our findings and demonstrate that T2DM patients present an impaired adaptability to physical stress (i.e., skeletal geometry) [39]. In addition, evidence demonstrates that T2DM patients present marked alterations in the cellular and physiological functioning of different tissues (e.g., brain, skeletal muscle) with a key role in the adaptive response to physical exercise [14–19].

Improvements in the cognitive function after ET, for example, are product of several mechanisms elicited by each session of exercise, such as increase in blood flow [40, 41]. However, investigations with humans [16, 18] and animal models [17] have demonstrated that T2DM is associated with a decreased diameter of cerebral perforating and middle cerebral arteries, followed by an impaired cerebral blood flow, which would impair the increase in cerebral blood flow in response to a physical stimulus.

Furthermore, regarding the neuromuscular apparatus, Allen et al. [14, 15] observed that T2DM patients with diabetic peripheral neuropathy presented a small number of motor units in the lower limbs. In addition, the findings indicate an altered protein metabolism, in favor of protein breakdown, due to insulin resistance in T2DM patients [19]. Interestingly, improvements in muscle strength and power in response to ET occur as a product of muscle hypertrophy [22–24] and/or neuromuscular adaptations (e.g., increased neural recruitment) [22, 23, 42].

Therefore, these investigations suggest that our MCEP was not fully sufficient to counteract the* negative* environment (e.g., reactive oxygen species, inflammation, hyperglycemia, and insulin resistance) responsible for the impaired adaptability in response to the physical stimulus present in T2DM patients. Thus, since maximal walking speed, TUG, and TUGcog performances involve a larger number of physical capabilities (e.g., muscle strength and power, attention, and inhibition) and physiological structures than usual walking speed, these tests were not altered in response to the MCEP.

Lastly, the current study evaluated the hemodynamic parameters of older T2DM patients. At baseline, our findings suggest that volunteers were in a* prehypertensive* state, while the CG group presented* normal* blood pressure values. These findings are consistent with existing knowledge, because the prevalence of high blood pressure values is around 80% in T2DM patients, contributing substantially to the high cardiovascular risk observed in these patients [12, 43].

The MCEP was able to elicit a marked decrease in blood pressure values of T2DM patients. Other studies have observed a reduction in blood pressure values after MCEP in T2DM patients, and different magnitudes of change in SBP and DBP may be observed among the studies [28]. In fact, in the experiment performed by Baptista et al. (2017) [28], volunteers showed 15 mmHg decrease in SBP, while the patients of the present study demonstrated 4.5 mmHg decrease. On the other hand, a larger decrease in DBP was observed in the current trial (−10 mmHg) when compared to the abovementioned study (−6 mmHg) [28].

The inconsistencies among these findings could be a function of the differences in the design of the MCEP (e.g., frequency, volume, duration, and intensity), as well as the initial state of the volunteers. Accordingly, it is not possible to indicate the crucial factor that influenced the different declines observed in blood pressure values. However, it is worth mentioning that in the trial of Baptista et al. (2017) [28] hypertensive patients were included in the sample, which limit the comparisons, since patients with high blood pressure values present larger reductions in these parameters after different regimes of ET, including multicomponent exercise [27], than patients with low blood pressure values. Moreover, Baptista et al. (2017) [28] evaluated blood pressure through a nonblinded method, which is associated with a measurement bias.

Some limitations of the current study should be mentioned and addressed in future studies to a better understanding about the effects of MCEP in T2DM patients, such as the lack of information regarding the educational level of the volunteers, blood analyses of the participants, evaluation of other cognitive domains, other designs of multicomponent exercise, and a sedentary CG.

## 10. Conclusions

Data of the current study indicate that a 6-month MCEP may improve mobility and reduce blood pressure in T2DM patients. However, maximal walking speed, transfer capacity, and executive function were only improved in CG, indicating that T2DM may present impaired adaptability in response to physical stimulus.

## Figures and Tables

**Table 1:** Characteristics of the older adults according to T2DM.

Variable	CG (*n* = 72)	T2DM (*n* = 72)	*P*
Age (years)	64.4 ± 5.7	66.0 ± 3.2	0.15
Weight (kg)	71.2 ± 13.8	75.8 ± 12.3	0.04
Height (m)	1.58 ± 0.0	1.58 ± 0.0	0.84
BMI (kg/m^2^)	28.4 ± 2.2	30.3 ± 2.8	0.27
WC (cm)	97.5 ± 11.7	102.6 ± 10.7	<0.001
HC (cm)	104.4 ± 9.6	105.8 ± 13.7	0.07
WC/HC	0.93 ± 0.06	1.07 ± 0.99	0.09
NC (cm)	36.7 ± 3.6	38.0 ± 3.1	0.03

CG = control group; T2DM = Type 2 diabetes mellitus; BMI = body mass index; WC = waist circumference; HC = hip circumference; NC = neck circumference.

**Table 2:** Functional and cognitive parameters.

Variable	CG (*n* = 72)	T2DM (*n* = 72)
Sit-to-stand (repetitions)		
Pre	10.1 ± 1.5	10.6 ± 2.1
Post	10.3 ± 2.6	11.4 ± 5.8
ES	0.09 *(trivial)*	0.18* (trivial)*
Δ	2.0	7.5

One-leg stand (s)		
Pre	16.9 ± 21.0	15.9 ± 11.9
Post	11.8 ± 9.7	20.7 ± 11.1
ES	0.31 *(trivial)*	0.41 *(trivial)*
Δ	−30.1	30.2

Usual walking speed (m/s)		
Pre	1.2 ± 0.2	1.2 ± 0.2
Post	1.8 ± 0.4^b^	1.3 ± 0.2^b^
ES	1.89 *(moderate)*	0.50 *(small)*
Δ	50	8.2

Maximal walking speed (m/s)		
Pre	1.7 ± 0.2	1.8 ± 0.4
Post	2.8 ± 0.2^b^	1.7 ± 0.2
ES	2.5 *(moderate)*	0.31 *(trivial)*
Δ	38,4	−5.5

TUG (s)		
Pre	6.7 ± 1.1	7.3 ± 1.8^a^
Post	7.1 ± 1.4	7.4 ± 2.1
ES	0.31 *(trivial)*	0.05 *(trivial)*
Δ	6.0	1.3

TUG with a cognitive task (s)		
Pre	7.4 ± 1.3	8.0 ± 2.1^a^
Post	8.5 ± 2.0^b^	8.5 ± 2.7
ES	0.65 *(small)*	0.20 *(trivial)*
Δ	14.9	6.2

CG = control group; T2DM = Type II diabetes mellitus; TUG = Timed “Up and Go”; ES = effect size; ^a^*P* < 0.05 versus CG; ^b^*P* < 0.05 versus baseline.

**Table 3:** Hemodynamic parameters.

	CG (*n* = 72)	T2DM (*n* = 72)
SBP (mmHg)		
Pre	129.1 ± 18.0	135.5 ± 17.0^a^
Post	129.3 ± 20.4	129.3 ± 21.7^b^
ES	0.01 * (trivial)*	0.31 *(trivial)*
Δ (%)	0.1	4.5

DBP (mmHg)		
Pre	77.0 ± 11.0	78.2 ± 9.5
Post	73.8 ± 9.9	70.5 ± 11.6^b^
ES	0.30* (trivial)*	0.72 *(small)*
Δ (%)	−4.1	−10.2

MAP (mmHg)		
Pre	92.8 ± 16.9	97.3 ± 9.9
Post	89.3 ± 20.4	90.0 ± 14.0^b^
ES	0.18 *(trivial)*	0.60 *(small)*
Δ (%)	−3.7	−7.5

HR (bpm)		
Pre	77.1 ± 11.9	77.4 ± 12.8
Post	80.1 ± 14.1	77.6 ± 16.9
ES	0.22 *(trivial)*	0.01 *(trivial)*
Δ (%)	3.9	0.2

DP (mmHg *∗* bpm)		
Pre	9848 ± 2693	10527 ± 2412
Post	10040 ± 3177	10009 ± 2742
ES	0.06 *(trivial)*	0.20 *(trivial)*
Δ (%)	−2.0	−5.0

PP (mmHg)		
Pre	51.1 ± 15.8	57.2 ± 16.4
Post	53.5 ± 18.8	58.7 ± 15.1
ES	0.13* (trivial)*	0.09 *(trivial)*
Δ (%)	4.7	2.6

Data are presented as mean ± SD; SBP = systolic blood pressure; DBP = diastolic blood pressure; MAP = mean arterial pressure; HR = heart rate; DP = double product; PP = pulse pressure; ^a^*P* < 0.05 versus CG; ^b^*P* < 0.05 versus baseline; ES = effect size.
